# Point-of-care coagulometry in prehospital emergency patients – are international normalized ratios useful?

**DOI:** 10.1186/s13049-015-0186-z

**Published:** 2016-02-19

**Authors:** Manuel F. Struck, Peter Hilbert-Carius

**Affiliations:** Department of Anaesthesiology and Intensive Care Medicine, University Hospital Leipzig, Liebigstr. 20, 04103 Leipzig, Germany; Department of Anaesthesiology, Intensive Care and Emergency Medicine, Bergmannstrost Hospital, Halle, Germany

## Commentary

With great interest we read the recent article of Beynon et al. [1]. The authors aimed to assess the validity of a point-of-care (POC) coagulometer measuring international normalized ratios (INR) utilized in a prehospital setting. Indeed, there is a great potential for POC tests in emergency patients and only few studies have been conducted. Although the authors highlighted limitations of their own study and the relevance of INR itself, we feel that the study has further important limitations seriously restricting the implications. Most important, the validity of a test is defined as the degree to which an instrument measures the construct it purports to measure, and this objective was not defined [2]. A coagulopathy *in general* does not exist and prothrombin times (PT) are not established tests for a coagulopathy *in general*. Since INR was developed only to monitor long-term treatment of vitamin K antagonists (VKA), effects of other coagulation conditions, i.e., antiplatelet treatment, thrombocytopenia, hyperfibrinolysis or inherited platelet disorders can not be detected by INR measurements. A possible application for POC coagulometers in the prehospital setting is to assess INR in emergency patients treated with VKA. However, this issue was extensively studied in large (non-emergency) cohorts involving appropriate sample sizes and valid study designs [3, 4].

In the past decades, VKA accounted for the most prescribed anticoagulants worldwide in patients with atrial fibrillation and deep vein thrombosis. This practice is now under considerable change since non-vitamin K antagonist oral anticoagulants (NOAC) are available [5]. The dimension of this change is currently not predictable but it might replace common practice patterns in the future. An interesting objective would be to observe the sensitivity of POC coagulometers for detecting these direct oral anticoagulants, i.e., rivaroxaban, apixaban, edoxaban or dabigatran [6]. Diagnosis of NOAC activity is not reflected by standard coagulation parameters (PT using INR or Quick’s value, and partial thromboplastin time (PTT), respectively). Furthermore, patients under NOAC who sustain injuries and/or surgery are exposed to relevant risks of prolonged bleeding without abnormal standard coagulation parameters [6, 7]. This may potentially affect clinical practice in emergency medicine and attention of general practitioners.

We agree with the authors that the implementation of POC coagulometers might be feasible in pre-hospital emergency settings but the search for an easily available parameter enlightening the complexities of the entire coagulation system continues.

## Abbreviations

POC: Point-of-care
INR: International normalized ratio
VKA: Vitamin K antagonist
NOAC: Non-vitamin K oral anticoagulants
PT: Prothrombin time
PTT: Partial thromboplastin time
